# Costs and consequences of the Family Nurse Partnership (FNP) programme in England: evidence from the Building Blocks trial

**DOI:** 10.12688/f1000research.20149.1

**Published:** 2019-09-13

**Authors:** Kerry Bell, Belen Corbacho, Sarah Ronaldson, Gerry Richardson, Kerry Hood, Julia Sanders, Michael Robling, David Torgerson, Building Blocks Trial Group

**Affiliations:** 1 Health Sciences, University of York, UK, York, North Yorkshire, YO10 5DD, UK; 2 Centre for Health Economics, University of York, UK, York, North Yorkshire, YO10 5DD, UK; 3 Centre for Trials Research, Cardiff University, Cardiff, CF14 4YS, UK; 4 School of Healthcare Sciences, Cardiff University, Cardiff, CF14 4XN, UK; 5 Population Health Trials, Cardiff University, Cardiff, CF14 4ER, UK

**Keywords:** Randomised controlled trial, Cost-consequence analysis, Pregnancy in adolescence, Prenatal care, Maternal health, Home visiting

## Abstract

**Background: **The Family Nurse Partnership (FNP) is a licensed intensive home visiting intervention programme delivered to teenage mothers which was originally introduced in England in 2006 by the Department of Health and is now provided through local commissioning of public health services and supported by a national unit led by a consortium of partners. The Building Blocks (BB) trial aimed to explore the effectiveness and cost-effectiveness of this programme. This paper reports the results of an economic evaluation of the Building Blocks randomised controlled trial (RCT) based on a cost-consequence approach.

**Methods**: A large sample of 1618 families was followed-up at various intervals during pregnancy and for two years after birth. A cost-consequence approach was taken to appraise the full range of costs arising from the intervention including both health and social measures of cost alongside the consequences of the trial, specifically, the primary outcomes.

**Results**: A large number of potential factors were identified that are likely to attract additional costs beyond the implementation costs of the intervention including both health and non-health outcomes.

**Conclusion**: Given the extensive costs and only small beneficial consequences observed within the two year follow-up period, the cost-consequence model suggests that the FNP intervention is unlikely to be worth the substantial costs and policy makers may wish to consider other options for investment.

**Trial registration**: ISRCTN23019866 (20/04/2009)

## Introduction

The Building Blocks trial evaluated the Family Nurse Partnership (FNP) programme, an intensive, nurse-led home visiting programme for young, first time parents who live in areas with a low socio-economic profile. This programme was developed in the USA and was introduced in England by the Department of Health in 2006 with the aim of improving outcomes for health, wellbeing and social circumstances of young first-time mothers and their children. In October 2015 the FNP was transferred from NHS England to Local Authorities (LAs) and it is now provided in approximately 125 different LAs in England. The FNP programme was introduced to be an integral part of the progressive universalism approach recommended in The Healthy Child Programme (HCP). The HCP is delivered by the Family Nurse rather than by health visitors for women who enrol onto the programme.

Despite an extensive evidence base in the US, very little was known about the generalisability to a UK context which implements a very different health care model to that in the US. Hence, the Building Blocks (BB) trial was commissioned to evaluate the effectiveness and cost-effectiveness of the FNP intervention when delivered in a comprehensive publicly funded health care setting. The results from the effectiveness analysis showed no statistically or clinically significant difference associated with FNP for any of the four primary outcomes: smoking cessation (adjusted OR 0·90, 97·5% CI 0·64–1·2), birth weight (adjusted mean difference 20·75 g, 97·5% CI –47·73 to 89·23), second pregnancies within two years (AOR 1·01, 0·77–1·33), or child A&E attendances and admissions to hospital (AOR 1·32, 97·5% CI 0·99–1·76, p=0.03) ^1^.

NICE has long recommended the use of cost-utility analysis as the primary means of assessing cost-effectiveness where health is the sole or dominant benefit of influence. However, with the move to local authority responsibility in 2013, NICE broadened its approach to the appraisal of public health interventions to place more emphasis on cost-consequence and cost-benefit analyses to supplement the cost-utility analysis for complex interventions ^2^. Unlike the cost-utility analysis which reports only on the number and cost of QALYs gained, the cost-consequences analysis (CCA) is able to present disaggregated costs and a range of outcomes which allows readers to form their own conclusions on relevance and relative importance to their own decision making context ^3^. This is particularly valuable for evaluation interventions with an array of health and non-health benefits that cannot be measured in a common unit. CCAs are not restricted to a particular viewpoint hence decision makers can estimate the impact of their decisions on sectors beyond health, such as education and criminal justice ^4^. Furthermore, CCA is able to take many items into account that local authorities are likely to find important, including the trade-off between long-term goals and a paucity of short-run funding, and spill over effects into other areas of local government responsibility.

Given the complexity of the FNP programme and the diverse range of policy relevant outcome measures, a multi-faceted approach was taken to evaluate the programme’s cost-effectiveness. A cost-utility analysis conducted alongside the trial showed only nominal benefits in terms of maternal QALY gains which were not considered cost-effective based on NICE (National Institute for Health and Care Excellence) acceptability thresholds ^5^.

The analysis aims to list all relevant health and non-health related resource use, largely public or third sector providers of universal and specialist services locally accessible to teenagers of young children, and the costs associated with each of the trial arms as well as the consequences of the trial, specifically, the primary outcomes. In the UK, health care policy decisions are largely driven by the National Institute for Health and Care Excellence (NICE) which base their recommendations on the number of quality adjusted life years (QALYs) that can be gained by an intervention. As the children in the participating families were too young to draw QALY estimates, the overarching perspective of the wider economic analysis that was conducted alongside the trial could only be completed from the perspective of the mother due to availability of data. In keeping with this approach, the cost-consequence analysis also considered only resource use of the mother.

## Methods

### Sample

The Building Blocks trial was a two-arm pragmatic, non-blinded, parallel-group, randomised controlled trial (RCT) which recruited within a community midwifery setting at 18 partnerships between LAs and primary and secondary care organisations in England. A total of 1645 participants were randomised in the study. The present analysis is based on the 1618 participants recruited to the trial that did not later become ineligible (e.g. due to miscarriage) or withdraw their consent. Participants in the Intervention arm (n=808) received home visits from the FNP nurse during their pregnancy and in the two years following childbirth. Participants allocated to the usual care arm (n=810) received care from the local maternity and health visiting services in line with usual practice. Details on recruitment and follow-up rates are described in full elsewhere ^1^. Informed consent was obtained from all individual participants included in the study.

### Outcome measures

The primary outcome measures for the trial were tobacco use at late pregnancy (34–36 weeks’ gestation), birth weight, emergency attendances and hospital admissions for the infant within 24 months of birth, and the proportion of women with a second pregnancy within 24 months post-partum. There were also a large number of secondary outcomes across a number of domains; socio-economic, maternal health and well-being, health behaviours, pregnancy and birth, social support and the use of services. For all primary outcomes a 97.5% confidence interval (CI) and p-value was presented; for all secondary outcomes a 95% CI and p-value was presented. The outcome measures are described in full elsewhere ^1^.

### Economic data collection

Data for outcomes and resource use were collected by self-reported questionnaires at various time-points throughout the trial, specifically; baseline, late pregnancy (34–36 weeks gestation) and 6, 12, 18, and 24 months postpartum. Baseline and 24 month data were collected by face-to-face interview by a locally based researcher whilst data at other time points were collected via telephone by qualified telephone interviewers. Additionally, data related to the use of health care services for each trial participant were collected from Hospital Episode Statistics (HES) via NHS Digital and primary care (general practitioner (GP) records). Birth data were collected by records abstraction from maternity records.

### Resource use

The mean numbers of the health and non-health care items per woman are presented for both groups for the duration of the trial in their respective units, e.g. mean number of GP visits, average number of weeks in education etc. The difference between the two groups for these items is also presented. The totals for each item (e.g. total number of GP visits) have been calculated for each participant in both groups over the duration of the trial (i.e. from baseline up to the 2-year interview).

Resource use is averaged across all women in each arm leading to small mean numbers for resource use. Median, minimum and maximum values are reported to highlight the highly skewed nature of resource use. Costs were largely associated with only a minority of women using each resource. Resource use and associated costs are reported in terms of the mother only. All consequences of the intervention, for both mother and child, are reported.

### Unit costs

Unit costs applied to the resource use were retrieved from several sources. Unit costs pertaining to health care resource use ( Table 1) were sourced from NHS reference costs ^6^ and Personal Social Service Research Unit (PSSRU) Unit Costs of Health and Social Care 2013 ^7^. However, as no unified document currently exists to provide unit costs for the non-health related items, these were sourced from other relevant sources such as governmental websites, research documents and reports. Given that costs were taken from a range of sources the years of pricing vary for each item. These are reported in Table 2. No attempt was made to discount/ inflate these items to a consistent rate as appropriate inflation rates for this type of resource are not available. It is thus acknowledged that current figures per resource use may differ slightly from those documented.

**Table 1.** Unit costs for health resource use.

Item	Unit	Cost	Reference	Notes
**GP**	Per Surgery consultation lasting 11.7 min Per out of surgery (home visiting) lasting 23.4 min	£45 £114	Unit Costs of Health and Social Care 2013	Including direct care staff costs & qualifications
**GP Nurse**	Per Surgery consultation lasting 15.5 minutes Per home visiting lasting 23.4 min	£13.4 £27.3	Unit Costs of Health and Social Care 2013	Assume same duration than GP home visit
**Midwife**	Antenatal visit (Community) Postnatal visit (Community) Home visit Midwife episode	£51 £68 £70 £65	NHS reference costs 2012/2013 (NHS trusts and NHS foundation trusts) Unit Costs of Health and Social Care 2013	Community Health Services – Health Visiting and Midwifery Total Outpatient attendances data
**Health visitor**	Per hour Per hour of home visiting	£49 £71	Unit Costs of Health and Social Care 2010	Assume same duration than GP home visits
**Counsellor**	Surgery consultation	£58	Unit Costs of Health and Social Care 2013	
**Mental health**	Per hour per team member	£36	Unit Costs of Health and Social Care 2013	Community mental health team for adults with mental health problems.
**Crisis** **Resolution team**	Per hour per team member	£37	Unit Costs of Health and Social Care 2013	
**Support worker**	Per hour	£22	Unit Costs of Health and Social Care 2013	
**Social worker**	Per hour	£79	Unit Costs of Health and Social Care 2013	
**Physiotherapist**	Surgery session per hour Hospital session per hour	£34 £36	Unit Costs of Health and Social Care 2013	
**FNP Supervisor** **Nurse**	Clinic or phone visit per minute Home visit per minute	£1.34 £1.62	Unit Costs of Health and Social Care 2013	Qualified nursing, midwifery & health visiting staff by Agenda for change band 8a, NHS England. Ratio of direct time on: Home visits (1:0.45) Patient work (1:0.20)
**FNP Nurse**	Clinic or phone visit per minute Home visit per minute	£1.17 £1.41	Unit Costs of Health and Social Care 2013	Qualified nursing, midwifery & health visiting staff by Agenda for change band 7, NHS England. Home visits (1:0.45) Patient work (1:0.20)

^ PSSRU unit costs are based on mean full-time basic salary; including overheads and qualifications. FNP - Family Nurse Partnership, PSSRU - Personal Social Service Research Unit

**Table 2.** Unit costs for non-health resource use.

Non-health related resource use				
*Education*				
Mainstream school or further education (FE) college	Per week of education	£77	The Centre for Social Justice (Press release, 2011)	Cost of mainstream education cited at £4000 per child per year. This was divided by 52 to give an estimate of weekly rate
Learning support unit (LSU)	Per week of education	£40 (+£77)	North Lincolnshire Learning Support Unit, 2012 ( http://shareit.yhgfl.net/nlincs /cpd/ ?page_id=2159)	Cost per session of 1 centre. Assumed mothers would have at least 1 session per week in addition to regular schooling
Pupil referral unit (PRU)	Per week of education	£346.15	The Centre for Social Justice (Press release, 2011)	Cost of PRU education cited at £18,000 per child per year. This was divided by 52 to give an estimate of weekly rate
Teenage mums support unit (TMSU)	Per week of education	£167.50 (+£77)	Government published information published online May, 2014 ( https://www.gov.uk/16-to- 19-education-financial-support-for-students)	Education charged at the same rate as mainstream schooling. Mothers in a TMSU are eligible for ‘Care to Learn’ funding which provides a bursary of £160-£175 per week (average £167.50)
*Other supportive services*				
Connexions advisor	Cost per hour	£38.50	Unit Costs of Health and Social Care 2013	Page 217 £847 per 22 hours of a connexions advisors time gives an hourly rate of £38.50 per hour
School nurse	Unit of activity	£27	Unit Costs of Health and Social Care 2013	Average UK unit cost of school based children’s health services
Young people centre/ youth service	Unit of activity	£17	The cost of providing street based youth work in deprived Communities, 2004 (Joseph Rowntree Foundation Report)	Cost per contact of youth services project
Children’s centre	Unit cost per hour	£64.25	Cost Effectiveness in Sure Start Local Programmes: A Synthesis of Local Evaluation Findings, 2005	Unit cost of Sure Start children’s centres
Child development centre	Unit cost per hour	£34	Unit Costs of Health and Social Care 2013	Unit cost for key worker
Crèche/ day nursery	Cost per hour	£4.28	Range of current sources, 2014	Average cost across a range of crèche/ day nursery facilities
Toddler group	Unit cost per hour	£5	Unit Costs of Health and Social Care, 2005	Page 26, Unit cost of toddler group
Leaving care services	Cost per hour	£20	Young People Leaving Care: A Study of Costs and Outcomes, 2006	Page 175, Leaving care worker/ personal adviser (per hour of client related activity)
Youth offending team	Unit cost per hour	£29	Unit Costs in Criminal Justice (UCCJ), 2013	Page 65, Cost of Youth Offending Team (YOT) practitioner
Social worker	Unit cost per hour	£57	Unit Costs of Health and Social Care 2013	Page 197, Unit cost per hour of social worker
*Childcare*				
Crèche/ day nursery at school or college	Cost per hour	£4.28	Range of current sources, 2014	Average cost across a range of crèche/ day nursery facilities
Nursery group at children’s centre	Cost per hour	£4.40	Childcare Costs Survey, 2014	Page 4, Average UK cost of 25 hours per week £109.89 which equates to £4.40 per hour
Child-minder	Cost per hour	£3.99	Childcare Costs Survey, 2014	Page 4, Average UK cost of 25 hours per week £99.77 which equates to £3.99 per hour
Any other childcare	Cost per hour	£3.82	Childcare Costs Survey, 2014	Page 4, Mean cost of all types of childcare
*Foster care (weeks)*				
Foster care mother	Unit cost per week	£636	Unit Costs of Health and Social Care, 2013	Page 88, Unit cost per week of foster care inclusive of admin and social services
*Temporary accommodation*				
B&B	Cost per week	£334.95	Immediate costs to government of loss of home, 2012	Page 4, Average cost per week, UK
Teenage parent accommodation	Cost per week	£151.78	Young people and teenage parent accommodation based services and floating support services, 2010 Supported Housing Commissioning Strategy - 2005-2010, 2005	Average of 2 sites taken: Page 4, Average cost per week (Brent, London) Page 29, Average cost per week (Sheffield)
Supported accommodation	Cost per week	£150	Supported lodgings as a housing option for young people report, 2008	Page 32, Average cost per week, UK
Mother and baby hostel/unit	Cost per week	£230	Christian Family Concern Mother & Baby Unit, 2014 ( http://www.familyspacecroydon.co.uk/ Contacts /details/christian-family-concern- mother-baby-unit/)	Actual cost per week at 1 site in London
Women’s refuge	Cost per week	£421.15	The Cost of Domestic Violence, 2004	Page 76, Average of UK women’s refuges
Homeless hostel	Cost per week	£107.45	Immediate costs to government of loss of home, 2012	Page 4, Average cost per week, UK

### Missing data

For the purpose of the cost-consequence analysis, multiple imputation analysis was considered unnecessary as the model serves only to highlight likely resource use and the average costs per resource. This decision was further vindicated by the small number of women who reported using each of the resources, which in some cases were less than five women. Such small numbers of affirmative responses would prevent imputations from running effectively and result in high error rates. Instead, several assumptions were made to reduce the amount of missing data. Where a particular answer to a question meant that sub-questions were skipped, a value of zero was attributed to the sub-questions. For example, respondents were first asked a yes or no question as to whether they had utilised a particular resource, then how often following an affirmative response. Where respondents stated that they had not used a resource, frequency of use was assumed to be zero. Conversely, where a positive response was noted but not frequency value was given, a value of one was assumed as a conservative estimate. Due to the high number of variables and low number of complete cases, it was not considered pragmatic to summarise each variable by complete case and adjusted values.

### Statistical analysis

The mean resource use was calculated for each resource use item according to the appropriate unit (e.g. mean number of days). The incremental difference between trial arms was calculated for each item of resource use by subtracting the values of participants allocated to receive usual care from the values of participants who were allocated to receive the intervention (FNP). Mean unit costs were calculated by item for each of the trial arms by multiplying the resource use by an appropriate unit costs (see Table 3 for unit costs). Resource use costs were summed by type, e.g. health related resource use or non-health related resource use. All analyses were conducted in Stata version 12 ^8^. The consequences presented in Table 3 are the primary outcomes of the Building Blocks trial and full details of how these were calculated are presented in primary trial paper ^9^.

**Table 3.** Cost-consequence balance sheet.

Costs (resource use)	Mean resource use per participant			Mean cost per participant		
	FNP	Usual care	Incremental (FNP-Usual Care)	FNP	Usual care	Incremental (FNP-Usual Care)
**Health related resource use**						
*Inpatient attendances (Length of stay/ number of day admittances)*						
Inpatient stay	3.99	4.09	-0.10	6354.58	6661.18	-306.60
Day admittances	3.53	3.58	-0.05	775.22	781.73	-6.51
*Outpatient attendances (Number of attendances)*						
Maternity services	7.31	7.13	0.17	733.13	716.18	16.95
Other attendances	1.31	1.42	-0.11	156.37	161.23	-4.86
*Hospital-related resource use (Number of attendances)*						
A&E visit	6.50	9.00	-2.50	167.07	172.79	-5.73
*Community based resources use (Number of clinic attendances/ home visits)*						
Midwife visits	10.40	10.69	-0.28	622.91	643.87	-20.97
Health visitor visits	5.88	12.93	-7.05	135.68	217.78	-82.10
*GP visitation (Number of visits)*						
Surgery visits	9.36	8.46	0.90	421.35	380.54	40.80
Home-based visits	0.21	0.21	0.00	24.31	24.45	-0.14
*Nurse visitation (Number of visits)*						
Surgery visits	2.07	2.20	-0.13	21.10	22.40	-1.30
**Non-health related resource use**						
*Education*						
Mainstream education	6.90	6.62	0.28	710.36	682.15	28.21
Learning support unit	0.26	0.19	0.07	10.40	7.75	2.65
Pupil referral unit	0.00	0.01	-0.01	0.00	2.88	-2.88
Teenage mother support unit	0.26	0.29	-0.03	67.63	79.15	-11.52
*Other supportive services (Number of contacts)*						
Connexions advisor	1.51	1.38	0.13	58.13	53.23	4.90
School nurse	0.04	0.04	0.00	3.41	4.10	-0.69
Youth services	0.16	0.23	-0.07	2.76	3.84	-1.08
Family information service	0.08	0.13	-0.05	2.65	4.45	-1.80
Children's Centre	2.31	1.96	0.35	148.30	125.64	22.66
Child development centre	0.02	0.10	-0.08	0.84	3.23	-2.39
Crèche/day nursery	0.48	0.49	-0.01	2.04	2.09	-0.05
Toddler group	0.93	0.83	0.10	4.63	4.17	0.46
Leaving care service	0.08	0.06	0.02	1.53	1.28	0.25
Fostering services	0.01	0.05	-0.04	0.52	1.89	-1.37
Youth offending team	0.05	0.01	0.04	1.51	0.18	1.33
Social worker	0.72	0.64	0.08	28.86	25.58	3.28
Addiction support unit	0.01	0.01	0.00	0.35	0.35	0.00
*Childcare (Days per week)*						
Crèche / Day nursery at school or college	1.35	1.10	0.25	5.79	4.71	1.08
Nursery group at Children’s Centre	0.38	0.25	0.13	1.68	1.09	0.59
Child-minder	0.46	0.34	0.12	1.85	1.35	0.50
Any other childcare	0.88	0.77	0.11	3.38	2.96	0.42
*Foster care (Weeks)*						
Mother	0.10	0.18	-0.08	60.61	114.64	-54.03
Child	0.18	0.17	0.01	117.28	106.00	11.28
*Housing (Weeks)*						
B&B	0.22	0.26	-0.04	75.03	87.67	-12.64
Teenage parent accommodation	0.74	0.36	0.38	146.21	70.48	75.73
Supported accommodation	0.75	0.39	0.36	112.13	57.78	54.35
Mother & baby hostel/unit	1.07	0.89	0.18	246.51	204.44	42.07
Women’s refuge	0.04	0.11	-0.07	15.12	46.27	-31.15
Homeless hostel	0.34	0.36	-0.02	36.57	38.34	-1.77
**Consequences**						
Smoking (smoking or non-smoking in late pregnancy)				OR: 0.9 [97.5% CI: 0.67 to 1.22] p = 0.51		
Birth weight (mean difference in grams)				Mean adjusted difference = 20.75g [97.5% CI: -39.13 to 80.64] p = 0.497		
Child A&E attendances and admissions (attendance/admission or no attendance/admission)				OR: 1.32 [97.5% CI: 1.02 to 1.70] p = 0.033		
Short duration to subsequent pregnancy (<2years) (pregnancy or no pregnancy)				OR: 1.01 [97.5% CI: 0.80 to 1.28] p = 0.920		

FNP - Family Nurse Partnership

## Results

Unit costs of all health and non-health related resources used by the mother only are presented in Table 1 and Table 2. The results of the cost-consequences analysis can be seen in Table 3. For each cost item, the mean resource use per participant for both the FNP arm and the usual care arm are displayed, alongside the incremental resource use. The mean cost per participant is also shown for each item according to trial arm, as well as the incremental cost. In terms of the consequences, the primary outcomes of the Building Blocks trial are listed for both the FNP arm, usual care arm and the difference between the two. A descriptive summary of the costs and consequences is provided, whereby items are discussed in a disaggregated format for the broad range of costs and consequences (spanning health care and non-health care) included in the analysis.

### Costs

Little difference was observed in terms of average health care resource use across the majority of services though consistently small differences were found between trial arms in favour of FNP. The largest differences in terms of resource use were the average number of A&E attendances and the mean number of health visitor visits received, both of which, as anticipated, were lower for women receiving the FNP intervention compared to women under usual care.

As only small differences in average resource use were observed, the average per woman spend on each resource reflected only slightly notable differences. Overall, average total health-related resource use costs were slightly lower for the FNP arm compared to usual care. Lower average costs were found in the FNP arm in relation to inpatient care, outpatient attendances not related to maternity services, A&E attendances, and midwife and health visitor visitations. The largest difference in costs was found in relation to inpatient care requiring one or more nights in hospital. In this instance, the mean difference between arms was £307 with lower costs being associated with FNP recipients. A difference was also found between the average cost per participant in terms of community-based maternity and early years services, specifically health visitor and midwife visitation. On average £82 less was spent per woman receiving the FNP intervention compared to usual care for health visitor visits (both home and clinic based) whilst £21 less was spent per woman on midwife visits (both home and clinic based) reflecting a mean reduction in spend of just over £100 per woman on these key early years services. The number of health visitor visits received by women was lower in the FNP arm than the usual care arm as visits from FNP nurses were received in place of standard health visitor visits. Consequently, though the cost attributed to health visitor visits appears reduced, there is the additional cost of the FNP nurses. The cost of the health visitors provided in usual care would thus not be saved through the FNP intervention but would be spent instead on specific FNP trained nurses. Only minor differences were noted in terms of cost associated with inpatient day admittances not requiring an over-night stay (-£6.51), non-maternity based outpatient attendances (-£4.86), and A&E attendances (-£5.73), with all being in favour of FNP. Omitting the intervention costs, a total of £8, 872 an average was spent on health care for women receiving the FNP intervention compared to a total average of £9, 162 per woman in the usual care arm, a difference of £290 per woman between the two arms.

In addition to health care resource use, there was very little difference in the non-health care resource use between the two groups. The average cost of non-health related resource use for women receiving the FNP intervention was £1,866 compared to £1,738 for women under usual care, an average difference of only £128 per woman in favour of the control group. In general, reported uptake of all non-health related resources was fairly low in both groups as can be seen through the mean and median values presented in Table 3. For the majority of resource items, the median resource use for both groups is zero, confirming a low uptake. Mean values are thus influenced by only a minority of women.

Participation in education and training was also similar between groups for both mainstream schooling and dedicated support units with little difference in resource use or mean costs.

In terms of supportive services, children’s centres, such as Sure Start centres, were the most widely used service by both groups, though this averaged at only 2 visits per woman in each of the trial arms. Mean costs of children’s centre use was slightly higher in the FNP arm. This is likely a consequence of outlying values as the maximum number of visits was considerably higher in the FNP arm compared to usual care (38 and 29).

Resource use of childcare was found to be particularly low at all time-points and overall mean resource use per woman, within both groups, was particularly low. Slightly higher costs were found in FNP women in line with the marginally higher rates of resource use. The most widely used type of childcare was crèche/day nursery within a school or college though the mean number of weeks used throughout the follow-up period was very low at only 1.35 weeks for FNP women and 1.10 weeks for usual care women. As this resource use was low the total resource use was low resulting in a low average cost per woman of £5.79 per FNP woman and £4.71 per usual care woman though the cost for women actually using childcare would be much higher.

Resource use for foster care and temporary accommodation was also low in terms of the number of women reporting usage of these resources for both groups of women. Average costs are thus driven by a minority of women. Average costs for temporary accommodation were higher for FNP women than usual care women for teenage parent accommodation, supported accommodation and mother and baby units due to increased use of these by FNP women. The largest average difference between groups was for teenage parent accommodation, with FNP women costing on average £75.73 more than usual care women; again this was due to higher levels of resource use in the FNP group.

### Consequences

***Primary outcomes***. No difference was observed in the rate of smoking in late pregnancy between the two arms (adjusted OR: 0.90, 97.5% CI: 0.64 to 1.28). There was no difference in the reported number of cigarettes smoked during late pregnancy for participants classified at baseline as smokers (adjusted difference in means, FNP-Usual Care: 0.119 cigarettes, 97.5% CI: -0.73 to 0.97). On average, birth weights were marginally higher in the intervention arm compared to the control arm though this was not statistically significant (adjusted difference in means, FNP-Usual Care: 20.75 grams, 97.5% CI: -47.73 to 89.23). There was no difference in the proportion of women reporting a second pregnancy within the two years of their first child’s birth (adjusted OR 1.01, 97.5% CI: 0.77 to 1.33). Rates of child emergency attendances and admissions for any reason in secondary care prior to their second birthday were higher in the intervention arm though the difference was not statistically significant (adjusted OR 1.32, 97.5% CI: 0.99 to 1.76). There were no differential effects due to age, deprivation, participation in employment, education or training, or basic life skills for any of the primary comparisons.

***Secondary outcomes***. Some benefit of the intervention was observed in terms of maternally rated language developmental delay in the children at both 12 (OR 0.50, CI: 0.35 to 0.72) and 18 months (OR 0.66, CI: 0.48 to 0.90). This was further reflected in the Early Language Milestone assessment at the end of the trial period which showed higher scores on average for children in the intervention arm (adjusted difference of means, 4.49, CI: 0.52 to 8.45) indicating more advanced levels of language development. Benefits of the intervention were also observed for breastfeeding intentions (adjusted OR 1.32, CI: 1.02 to 1.70) though this did not translate into a difference in the actual proportions of mothers initiating breastfeeding between the two arms.

A greater proportion of children in the intervention arm attended an Emergency Department for an injury or ingestion at 6 months (adjusted OR 1.52, CI 0.86 to 2.70) and 12 months (adjusted OR 1.16, CI 0.92 to 1.46), were referred to social services by two years (adjusted OR 1.27, CI: 0.93 to 1.73) and had a safe guarding event recorded in their GP record (adjusted OR 1.85, CI: 1.02 to 2.85) compared to children in the control group. These differences were not statistically significant.

No evidence of a difference was observed for any other maternal or parenting and child outcomes assessed in the trial.

At two years, mothers in the intervention arm reported lower rates of not being in employment, education or training (NEET) than the control arm, though this was not statistically significant. A larger proportion of participants in the intervention arms reported a maximum level of social support at 18 months postpartum (25.7%) compared to those in the control arm (20.3%) with a similar difference being maintained at 24 months. A small difference between the arms was observed over the whole follow-up period (adjusted OR 1.5, CI: 1.06 to 2.12). Fewer participants in the intervention arm reported ever being homeless during the study period compared to the control arm (adjusted OR 0.76, CI: 0.55 to 1.05) and higher levels of self-efficacy were observed in the intervention arm (adjusted mean difference 0.44, CI: 0.10 to 0.78).

No evidence of a difference was observed for any other parental life course outcomes assessed in the trial.

## Discussion

This analysis shows limited differences between the resource uptake and associated costs between the two trial arms (FNP intervention and usual care). As demonstrated in Table 2, for most of the health care resource use items, average costs were slightly lower for the FNP arm compared to usual care. Conversely resource use and associated average per person costs were higher for non-health related items. This however is not necessarily a negative outcome as some of the items associated with greater costs were positive aspects such as attendance at a mainstream education programme and attendance at children’s centres. Despite these resources being considered positive outcomes it is important to remember that these are still additional costs which could be incurred as a result of the FNP intervention and should be considered in policy making. It is also worth noting that although there were more child A&E attendances and admissions associated with families receiving the intervention, this is unlikely to be a detrimental effect of the intervention. Instead this probably reflects an increased awareness of mothers receiving the intervention, and their increased contact with a health professional could mean that health concerns are identified more readily causing mothers to seek more help.

Previous studies of the FNP programme in the United States (US) have shown positive results in terms of improved prenatal health, childhood injuries, subsequent pregnancies, maternal employment and child school readiness ^10– 12^. The Building Blocks study is the first randomised controlled trial of FNP in the UK. The statistically insignificant effects observed in the present work which are discussed in the effectiveness paper ^9^ likely reflect the differences in health care provision between the US and the UK. In the US, low income mothers are not able to access many health care and social services. In contrast, all pregnant women in the UK can access a wide provision of maternal care including community care family doctors, midwives and specialist public health nurses. It is thus likely that the FNP programme does not offer a sufficiently enhanced service to the level of usual care provided in the UK. This study thus adds to the knowledge base of FNP and is useful to policy makers considering the value of implementing FNP within a UK-style health care system.

As a methodological approach, cost-consequence analysis is generally limited by its dependence on subjective decision making by policy makers, as it relies on individual judgement of what consequences are sufficiently beneficial to merit investment. The lack of a comparative statistical component could also be viewed as a limitation. However, the cost-consequence model is of value to provide a simplistic overview of costs associated with the intervention, particularly those that are not obvious such as youth offending services and family information services. A cost-consequence balance sheet was implemented to provide policy makers with a clear descriptive summary of the health and non-health related costs associated with the FNP intervention. This offers an advantage over more technical methods of economic analysis. Furthermore, though the use of QALYs is generally favoured by clinical decision making bodies such as the National Institute for Health and Care Excellence (NICE) ^13^, the calculation of QALYs may not always be sensitive in particular populations. When QALY gains were assessed in a cost-utility analysis conducted as part of the BB trial, no significant gains were identified and the intervention was deemed not cost-effective when considered from the perspective of the mother ^14^. Given the dimensions of the EQ-5D (pain, mobility, self-care, usual activities, and depression/anxiety) it may be the case that the instrument was insensitive to the population and the intentions of the intervention to primarily improve child health outcomes rather than maternal health outcomes thus giving further weight to the case for considering outcomes beyond health.

The analysis considered only resource use of the mother; this is due to the overarching perspective of the wider economic analysis that was conducted alongside the trial, the primary analysis being a cost-utility analysis. Currently there is no accepted instrument for measuring utility in children as young as those in the Building Blocks trial hence the cost-utility analysis could only take the perspective of the mother. Future research could incorporate child resource use but given the absence of statistically significant results for any of the Building Blocks trial primary outcomes, this would not change the overall message of the work and hence was deemed unnecessary.

As an approach, cost-consequence analysis cannot usually conclude whether an intervention is worth funding as the judgement usually lies upon the gains that would likely be attained by recipients. However, given that the FNP intervention does not bring about any significant improvements to recipients compared to usual care on the basis of the short-term outcomes, together with the higher cost of delivering the intervention compared to usual care (£1812 more per woman), these two factors suggest that based on current evidence the continuation of the programme cannot be justified.

In all, the cost-consequence model summarises the between-group differences in resource use and costs, alongside the primary trial outcomes. As can be seen, there are a large number of potential factors that are likely to attract additional costs beyond the obvious implementation costs of the intervention. Given these costs and the way in which only small benefits were observed in the trial analysis when considering primary outcomes alone, the cost-consequence model provides further support that the FNP intervention is unlikely to be worth the substantial costs and policy makers may wish to consider other options for investment.

## Ethical approval

The trial is registered with International Standard Randomised Control Trial Number ISRCTN23019866 on 20 April 2009. The Building Blocks trial protocol and all amendments were reviewed and approved by the Wales NHS Research Ethics Committee (09/MRE09/08). All procedures were in accordance with the ethical standards set out in the 1964 Helsinki declaration and its later amendments.

## Data availability

### Underlying data

The Building Blocks Trial dataset comprises data from multiple sources including third party. The Hospital Episode Statistics (HES) data, which forms a substantial component of the present analysis, is supplied under licence from NHS Digital. The HES dataset is not currently available for analysis to researchers outside Cardiff (the lead institution) due to the information provided to participants at consent (which identified which institutions were acting as data controller / processors).

Researcher can apply for access to the full dataset by contacting Cardiff Centre for Trials Research (CTR) (see: https://www.cardiff.ac.uk/centre-for-trials-research/about-us/data-requests). The restriction on HES data (originally supplied via NHS Digital) applies. CTR will consider whether section 251 approval would enable release of participant identifiers to apply directly to NHS Digital for the data used in the trial.

### Extended data

The results of the Building Blocks trial are presented in full at: https://www.cardiff.ac.uk/__data/assets/pdf_file/0009/504729/Building-Blocks-Full-Study-Report.pdf

This publication includes the full CONSORT statement for the trial on page 564.
