# Supplementary material for: Costs and consequences of the Family Nurse Partnership (FNP) programme in England: evidence from the Building Blocks trial
Source: F1000Res. 2019 Sep 13;8:1640. [Version 1] doi: 10.12688/f1000research.20149.1 (PMC6784875; doi:10.12688/f1000research.20149.1)
Supplement: Peer review history

# Peer review: Reviewer response for version 1

**DOI:** 10.5256/f1000research.22134.r53943

**Published:** 2019-10-08

**Authors:** Jacqueline Barnes

**Affiliations:** 1 Department of Psychological Sciences, Birkbeck, University of London, London, UK

The Building Blocks team are to be congratulated for taking a cost-consequences approach (CCA) rather than the cost utility method relying on QALYs, the strategy frequently applied to interventions. The CCA is much more relevant for this type of trial, with many non-medical potential benefits included in the secondary outcomes. It is well known that the long-term impact of early intervention such as the Nurse Family Partnership (NFP, known as FNP in the UK) can be within the areas of education, employment or criminal justice. Unfortunately for the team they currently have information only up to the end of programme delivery when children were 24 months of age, and are reliant of the primary outcomes being medical in nature. Many of the most well documented impacts of the NFP intervention, from trials in the USA, have been identified when the children are in primary or secondary school, or during young adulthood. It is hoped that further follow-up of the trial participants will be feasible. The paper acknowledges this point.

 The design is strong, albeit limited due to lack of sufficient costing of services used by infants, meaning that all of the services used are those use by the mother. Thus it is not clear whether a visit to a children's centre includes any services explicitly experienced only by the child are covered. One point about the costs applied to Children's Centre visits is that they are based on the economic evaluation of Sure Start Local Programmes (SSLPs). While Children's Centres, where if they still operate, are largely derived from SSLPs, the range of services is likely to be fewer meaning that costs may be in fact lower.

 All the details of the method are clearly presented, meaning that the work could be reproduced if there was access to the full dataset. This is not possible since participant consent precluded anyone not a member of the Building Blocks team having access to the Hospital Episodes Statistics data. This is clearly stated in the paper.

 The discussion is very sensible, noting that aspects of the mothers' experiences such as attending an educational establishment or a children's centre, while incurring costs at the time are likely to lead to less of a cost to society in the future if this leads to better employment prospects, or a child more ready to start school. Similarly more women and infants gaining access to housing rather than being homeless is of great benefit to society and to local communities. Thus it is disappointing that the paper concludes that continuation of the programme cannot be justified. This strong conclusion is not really warranted until the children can be followed until the beginning of formal education at the very least. The FNP children had some benefits in terms of language development so it is important to identify whether fewer required the support of speech and language therapists or educational psychologists and special education teachers. Thus it would have been useful to have some provisos included in the conclusion. While it is not possible in relation to the Building Blocks RCT, it would also be useful to comment that further research with a sample of women identified using additional risk indicators beyond age and first pregnancy, and one involving FNP practitioners with substantial experience rather than newly trained Family Nurses, would add to the capacity for commissioners to decide about continuing to support the FNP programme.

I confirm that I have read this submission and believe that I have an appropriate level of expertise to confirm that it is of an acceptable scientific standard.

# Peer review: Reviewer response for version 1

**DOI:** 10.5256/f1000research.22134.r53944

**Published:** 2019-10-01

**Authors:** William Hollingworth, Shaun Harris

**Affiliations:** 1 Department of Population Health Sciences, Bristol Medical School, University of Bristol, Bristol, UK

The authors present a cost-consequences analysis of a family nurse (FNP) led intervention (versus usual care) to support teenage mothers in England. The analysis is based on approximately 1600 participants recruited to the Building Blocks RCT. The randomised design and size of the study should provide robust results for policy makers.

 Given the diverse range of costs and outcomes a cost consequence analysis is a reasonable and appropriate approach to take. The pragmatic approach to address missing data also appeared to be reasonable given the context. Key outcomes and cost-utility data from this trial have already been reported (Robling *et al.* 2016 ^1^; Corbacho *et al.* 2017 ^2^). Therefore, the contribution of this paper is largely in providing additional details about health (and wider) resource use in each arm of the trial.

 The results show that there is little difference in either resource uptake or associated costs between FNP and usual care. The authors conclude that the intervention is unlikely to be cost-effective. The authors suggest that previous positive results from US studies might not be replicated in England due to more extensive existing access to maternity care in the UK.

  There are several issues that should be clarified:

1. In Table 3, it would be useful to report the total healthcare costs to establish the aggregate cost differences (and 95% CI) between FNP and usual care. These total costs are briefly mentioned on page 9 (paragraph 2) but do not appear in Table 3.
2. The cost of the FNP intervention itself should be included in Table 3. These are only introduced in the discussion section currently.  Inclusion in Table 3 should allow the authors to estimate the net incremental healthcare costs of FNP.
3. Although there is no one agreed approach to inflating costs across difference sectors of the economy, the approach taken by the authors (of not standardizing cost years) seems unsatisfactory as it does not allow the estimation of total costs (healthcare and other sectors) that would be useful for readers/policy makers.  A GDP deflator index - while not perfect - would be a better starting point.
4. Page 9  (paragraph 3): median values are not presented in table 3.
5.  A brief description of the intervention theory and aims and the eligibility criteria would help this article 'stand alone' from previous articles.

We confirm that we have read this submission and believe that we have an appropriate level of expertise to confirm that it is of an acceptable scientific standard.

## References

[1] : Effectiveness of a nurse-led intensive home-visitation programme for first-time teenage mothers (Building Blocks): a pragmatic randomised controlled trial. *Lancet*.2016;387(10014) : 10.1016/S0140-6736(15)00392-X 146-55 10.1016/S0140-6736(15)00392-X 26474809PMC4707160

[2] : Cost-effectiveness of the Family Nurse Partnership (FNP) programme in England: Evidence from the building blocks trial. *J Eval Clin Pract*.2017;23(6) : 10.1111/jep.12799 1367-1374 10.1111/jep.12799 28799197
